# Idiopathic Small Bowel Diaphragm Disease Without Use of Non-steroidal Anti-inflammatory Drugs: A Case Report and Review of the Literature

**DOI:** 10.7759/cureus.32735

**Published:** 2022-12-20

**Authors:** Dhir Gala, Han Chen Tom Tsou, Josef Khoury, Mili Shah, Stephen Wonnacott, Carly M Funk, Vikash Kumar, Paul Toomey

**Affiliations:** 1 Internal Medicine, American University of the Caribbean, Brooklyn, USA; 2 Internal Medicine, Ross University School of Medicine, Barbados, BRB; 3 Internal Medicine, American University of the Caribbean School of Medicine, Brooklyn, USA; 4 School of Medicine, American University of the Caribbean, Brooklyn, USA; 5 Internal Medicine, Jinnah Sindh Medical University, Karachi, PAK; 6 Surgical Gastroenterology, Epsom and St Helier University Hospitals, London, GBR

**Keywords:** abdominal pain, iron deficiency, anemia, small bowel stricture, diaphragm disease

## Abstract

Small bowel diaphragm disease is a rare condition that is characterized by the presence of diaphragm-like strictures that causes intermittent or complete small bowel obstruction. Most cases are asymptomatic until presented with severe abdominal pain due to small bowel obstruction or diagnosed during anemia workup as a cause of occult gastrointestinal bleeding. Small bowel diaphragm disease is usually associated with long-term use of non-steroidal anti-inflammatory drugs (NSAIDs). Here, we present the case of a 50-year-old male with no history of NSAID use who presented with abdominal pain and iron deficiency anemia. He was postoperatively diagnosed with idiopathic small bowel diaphragm disease.

## Introduction

Small bowel diaphragm disease is a rare condition that is commonly associated with long-term use of non-steroidal anti-inflammatory drugs (NSAIDs). It causes intraluminal narrowing secondary to a series of repairs and injuries leading to the deposition of collagenous scars [1]. The strictures commonly occur in the midline and can lead to either intermittent or complete small bowel obstruction, as well as unexplained abdominal pain or occult gastrointestinal bleeding. Unfortunately, the progression of the disease does not stop with the discontinuation of NSAID use, and, therefore, definitive treatment often requires surgical resection [2].

While small bowel diaphragm disease more commonly affects the small bowel, it is worth noting that the colon can be affected as well [3]. Grossly, small bowel diaphragm disease shows ulceration on the diaphragm with serosa retractions of the small bowel causing a ring-like appearance resulting in stenosis of the lumen. Histopathologically, there is a wide variation of presentations but with a common theme of eosinophils in the mucosa [4]. Here, we present a case of small bowel diaphragm disease presenting with abdominal pain and iron deficiency anemia.

## Case presentation

In May 2020, a 50-year-old male presented with shortness of breath and chronic fatigue for the past few months. In addition, he had intermittent recurring hypogastric abdominal pain lasting about 5-10 minutes with occasional vomiting. The pain was colicky in nature. There were no identifiable triggers or positions that alleviated the pain. He was prescribed codeine, tramadol, and lansoprazole as needed for the abdominal pain. He was otherwise healthy with no chronic medical conditions or history of prior surgery and denied taking any other medications including NSAIDs. He denied any recent unintentional weight loss, hematochezia, constipation, or diarrhea.

On presentation, his temperature was 36.9°C, blood pressure was 129/73 mm Hg, heart rate was 91 beats/minute, respiratory rate was 18 breaths/minute, and oxygen saturation was 97%. Further, his labs showed iron deficiency anemia with hemoglobin of 9.1 g/dL, mean corpuscular volume of 78 fL, a red blood cell count of 3.97 million cells/µL, red cell distribution width of 15.4%, and ferritin of 6 µg/L. He was referred to the gastroenterology team to investigate the cause of his iron deficiency anemia. Endoscopy and colonoscopy were performed in June 2020, and the results were normal with no signs of ulceration, obvious blood loss, tumor, or malignancy.

**Figure 1:**
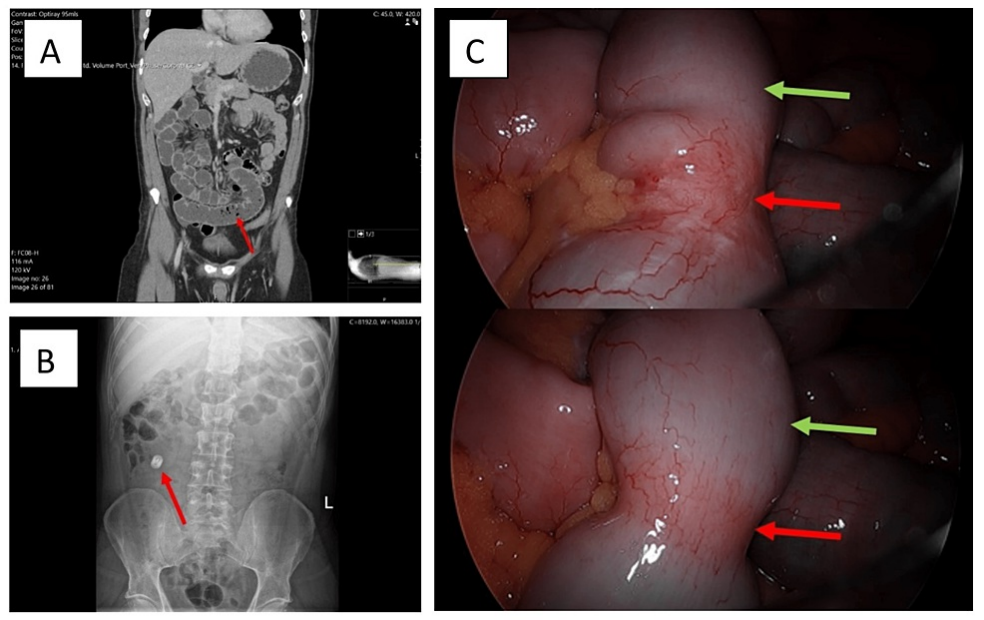
A. Coronal computed tomography of the abdomen scan showing signs of dilated small bowel and fecalization (red arrow), suggesting small bowel obstruction. B. Abdominal X-ray showing the patency capsule that did not pass through the small bowel (red arrow). C. Laparoscopy images showing macroscopic changes in the small bowel. Strictures (red arrows) and dilations (green arrows) are visible in both images.

Following this, a computed tomography (CT) scan of the thorax, abdomen, and pelvis was performed. The CT scan showed moderately dilated small bowel loops involving the distal jejunum and proximal ileum suggesting an incomplete closed-loop partial obstruction without an obvious cause (Figure 1, Panel A). Although these radiographic findings suggested possible obstruction, considering no prior history of surgery, the risk of adhesions was low. Based on the radiologic findings along with a negative endoscopy and colonoscopy, small bowel adenocarcinoma was considered as a potential differential diagnosis. To follow up on the CT scan, magnetic resonance imaging was performed in July 2020 which showed no small bowel lesions ruling out small bowel adenocarcinoma.

To treat his iron deficiency an iron tablet was prescribed. This was not well tolerated due to nausea and stopped soon after. From July 2020 to November 2020, his hemoglobin dropped from 9.5 g/dL to 7.0 g/dL. He was given two units of blood transfusion which raised his hemoglobin to 9.4 g/dL. A video capsule endoscopy was not performed because the patency capsule did not pass through as seen on an abdominal X-ray (Figure 1, Panel B). A positron emission tomography-computed tomography scan was then performed which showed no abnormal findings.

In March 2021, he continued to be anemic with a hemoglobin of 7.6 g/dL and complained of ongoing spasmodic abdominal pain. The pain was still colicky but had progressively worsened from the presentation. He reported poor appetite, early satiety, and nausea with no vomiting. In addition, he lost 21 pounds unintentionally in the last two months likely due to poor per oral intake. He also started noticing frank blood in his stool for the first time. Lastly, he described occasional nighttime leg “discomfort” with an urge to move, leading to sleep disturbances, which was likely restless leg syndrome secondary to chronic iron deficiency anemia. Two units of iron were then transfused ultimately raising his hemoglobin to 10.1 g/dL.

In April 2021, a laparoscopy with a careful inspection of the small bowel from the ileocecal valve to the duodenojejunal flexure was performed. The laparoscopy revealed three distinct areas of congested appearing, thickened, and narrowed small bowel within the ileum (Figure 1, Panel C) with associated mesenteric lymphadenopathy. The diseased segment of the small bowel with the associated mesenteric vessels was removed using Alexis wound protector-retractors. The remaining bowel was anastomosed using hand-sewn end-to-end anastomosis with Vicryl sutures. Histology of the resected small bowel showed ulceration with elongated mucosal folds and submucosal fibrosis consistent with the diagnosis of small bowel diaphragm disease.

Post-surgery, hospitalization was uneventful. He was discharged on postoperative day five. At the outpatient follow-up four weeks after the operation, he reported doing well. He did not have any abdominal pain, nausea, vomiting, or blood in his stool. His activity level was almost back to normal. Two months after the operation, his hemoglobin improved to 12.1 g/dL. At the eighth-month postoperative follow-up, he continued to be well without any abdominal symptoms with increased energy and improved hemoglobin in the normal range (Table 1). The shortness of breath and fatigue symptoms resolved. He also reported mild improvement in restless leg syndrome symptoms.

**Table 1:** Summary of laboratory values at presentation and two and eight months post-resection of the strictured small bowel. MCV = mean corpuscular volume; MCH = mean corpuscular hemoglobin; MCHC = mean corpuscular hemoglobin concentration; RDW = red cell distribution width; L = low; H = high

Lab results	At presentation (May 2020)	Two months postoperative (June 2021)	Eight months postoperative (December 2021)
RBC count (million cells/ µL)	3.97 (L)	4.78	4.95
Hemoglobin (g/dL)	9.1 (L)	12.1 (L)	13.5
Hematocrit (%)	0.308 (L)	0.392 (L)	0.424
MCV (fL)	78 (L)	82 (L)	86
MCH (pg)	22.9 (L)	25.3 (L)	27.3 (L)
MCHC (g/dL)	295 (L)	309	318
RDW (%)	15.4 (H)	17.3 (H)	15.8 (H)
Ferritin (µg/L)	6 (L)	13 (L)	21 (L)

## Discussion

Small bowel diaphragm disease is a rare condition. The diaphragm refers to rings of scar tissue that form a band around the bowel lumen [5]. The usual symptoms are non-specific such as vomiting and abdominal pain with underlying anemia. The most common presentation includes a history of NSAID use, anemia, and obstructive symptoms [6]. The most common manifestations of clinically significant small bowel diaphragm disease are gastrointestinal bleeding (72.9%), bowel obstruction (65.8%), weight loss (18.1%), and constipation (10.3%). In this case, the patient presented with iron deficiency anemia and hypogastric pain that did not align with any other conditions or chronic NSAID use. The diagnosis was achieved through laparoscopy with histology of the resected small bowel.

Imaging can potentially be used to diagnose small bowel diaphragm disease preoperatively. A case series showed that the most common CT findings included strictures, wall thickening, mucosal hyperenhancement, and bowel dilation. However, due to the unblinded nature of this study, the detection of the disease via imaging may have been over-reported [7]. Another technique used to diagnose small bowel diaphragm disease is the barium contrast study which shows the narrowing of the small bowel, but normal findings such as pilae circulares can resemble this as well, thus making it less effective [8]. Capsule endoscopy can also be used to visualize the strictures in the small bowel. Unfortunately, this method poses a major risk, possibly causing retention of the capsule and leading to complete bowel obstruction [7,8].

The best diagnostic and therapeutic intervention for small bowel diaphragm disease is laparoscopy which shows dilations and strictures in the affected bowel segment. It is worth noting that an absence of these macroscopic findings does not rule out small bowel diaphragm disease of the small bowel [9]. Additionally, an intraoperative enteroscopy can be performed to identify affected segments [8]. Definitive treatment consists of resection and strictureplasty of the affected small bowel, as was done in this case. In a study following patients postoperatively, there was no recurrence of symptoms at 10 months. Additionally, it is advised that all patients stop taking NSAIDs after the diagnosis is confirmed [10].

NSAIDs are one of the most widely prescribed drugs globally for a multitude of indications, including analgesia, heart disease, and rheumatological conditions [8,11]. As this condition has been reported in a bypassed segment of the bowel, the absence of direct mucosal contact does not preclude the formation of small bowel diaphragm disease, suggesting a more likely multifactorial mechanism of injury and systemic effect of NSAIDs [11]. NSAIDs reduce villous microcirculation, resulting in a reactive inflammatory process and increased mucosal permeability [11]. As a result, gut microbes and bile acids penetrate the mucosa leading to further inflammation and the formation of strictures and mucosal diaphragms [7,11]. In a capsule endoscopy study of 160 patients with chronic NSAID use (three months or longer duration), two patients had small bowel diaphragm disease. Further, 2% of participants were found to have diaphragm-like strictures with 3% having bleeding without an identifiable lesion [5].

## Conclusions

Small bowel diaphragm disease in the absence of long-term NSAID use is extremely rare. The most common presentation of clinically significant small bowel diaphragm disease is gastrointestinal bleeding, bowel obstruction, weight loss, and constipation. Non-specific presentations such as weight loss and constipation warrant the exclusion of other pathologic processes such as inflammatory bowel disease, coeliac disease, food intolerances, and bowel cancer. Unexplained abdominal symptoms should raise suspicion for small bowel diaphragm disease. CT scan can potentially show small bowel strictures or focal areas of stenosis and wall thickening. Surgeons can orient radiologists to focus their interpretation of scans on small bowel strictures and bowel wall thickening which may aid in diagnosis preoperatively. Small bowel diaphragm disease should be considered in the differential diagnosis of otherwise unexplained recurrent abdominal pain and iron deficiency anemia.

## References

[1] Going JJ, Canvin J, Sturrock R (1993). Possible precursor of diaphragm disease in the small intestine. Lancet.

[2] McNally M, Cretu I (2017). A curious case of intestinal diaphragm disease unmasked by perforation of a duodenal ulcer. Case Rep Med.

[3] Saleem N, Marella HK, Ali B, Tombazzi CR (2020). Colonic diaphragm disease secondary to nonsteroidal anti-inflammatory drug use. Proc (Bayl Univ Med Cent).

[4] De Petris G, López JI (2008). Histopathology of diaphragm disease of the small intestine: a study of 10 cases from a single institution. Am J Clin Pathol.

[5] Maiden L, Thjodleifsson B, Seigal A, Bjarnason II, Scott D, Birgisson S, Bjarnason I (2007). Long-term effects of nonsteroidal anti-inflammatory drugs and cyclooxygenase-2 selective agents on the small bowel: a cross-sectional capsule enteroscopy study. Clin Gastroenterol Hepatol.

[6] Slesser AA, Wharton R, Smith GV, Buchanan GN (2012). Systematic review of small bowel diaphragm disease requiring surgery. Colorectal Dis.

[7] Flicek KT, Hara AK, De Petris G, Pasha SF, Yadav AD, Johnson CD (2014). Diaphragm disease of the small bowel: a retrospective review of CT findings. AJR Am J Roentgenol.

[8] Wang YZ, Sun G, Cai FC, Yang YS (2016). Clinical features, diagnosis, and treatment strategies of gastrointestinal diaphragm disease associated with nonsteroidal anti-inflammatory drugs. Gastroenterol Res Pract.

[9] Chernolesskiy A, Lanzon-Miller S, Hill F, Al-Mishlab T, Thway Y (2010). Subacute small bowel obstruction due to diaphragm disease. Clin Med (Lond).

[10] Li F, Gurudu SR, De Petris G (2008). Retention of the capsule endoscope: a single-center experience of 1000 capsule endoscopy procedures. Gastrointest Endosc.

[11] Pereira R, Slater K (2019). Small bowel diaphragm disease from long-term non-steroidal anti-inflammatory use. BMJ Case Rep.

